# Enrichment Effects of Gestures and Pictures on Abstract Words in a Second Language

**DOI:** 10.3389/fpsyg.2017.02136

**Published:** 2017-12-15

**Authors:** Claudia Repetto, Elisa Pedroli, Manuela Macedonia

**Affiliations:** ^1^Department of Psychology, Università Cattolica del Sacro Cuore, Milan, Italy; ^2^Applied Technology for NeuroPsychology Lab, Istituto Auxologico Italiano, Milan, Italy; ^3^Information Engineering, Johannes Kepler University Linz, Linz, Austria; ^4^Max Planck Institute for Human Cognitive and Brain Sciences, Leipzig, Germany

**Keywords:** gestures, abstract words, second language learning, enactment effect, embodied cognition

## Abstract

Laboratory research has demonstrated that multisensory enrichment promotes verbal learning in a foreign language (L2). Enrichment can be done in various ways, e.g., by adding a picture that illustrates the L2 word’s meaning or by the learner performing a gesture to the word (enactment). Most studies have tested enrichment on concrete but not on abstract words. Unlike concrete words, the representation of abstract words is deprived of sensory-motor features. This has been addressed as one of the reasons why abstract words are difficult to remember. Here, we ask whether a brief enrichment training by means of pictures and by self-performed gestures also enhances the memorability of abstract words in L2. Further, we explore which of these two enrichment strategies is more effective. Twenty young adults learned 30 novel abstract words in L2 according to three encoding conditions: (1) reading, (2) reading and pairing the novel word to a picture, and (3) reading and enacting the word by means of a gesture. We measured memory performance in free and cued recall tests, as well as in a visual recognition task. Words encoded with gestures were better remembered in the free recall in the native language (L1). When recognizing the novel words, participants made less errors for words encoded with gestures compared to words encoded with pictures. The reaction times in the recognition task did not differ across conditions. The present findings support, even if only partially, the idea that enactment promotes learning of abstract words and that it is superior to enrichment by means of pictures even after short training.

## Introduction

Despite progress in second language (L2) instruction, vocabulary learning remains a task that people still accomplish by reading bilingual word lists and by repeating them until the words are memorized, as described by Oxford and Crookall (1990). One way to make learning faster and more resistant to decay has proven to be enrichment: novel words are accompanied by additional sensory information such as pictures or pantomimes. This is the principle on which mnemonic strategies have been based for centuries (Riesenberg et al., 2009). Shams and Seitz (2008) suggest that our brain is tuned to learn and to operate in multisensory environments. Therefore, multisensory learning can better simulate natural settings and lead to superior memory performance compared to unimodal learning (reading or listening). Better retrievability of enriched words can be accounted for by the Connectivity Model (Klimesch, 1987). It proposes that a concept is represented as a network of interconnected nodes – the features. The model assumes that the more the nodes are interconnected, the faster the information is processed and retrieved within the network. If a word in L2 (i.e., a string of phonemes) is enriched by a picture or other sensory information, the network grows larger. Complexity enhances information processing, and consequently memory and speed of retrieval (Klimesch, 1994).

### Enriching L2 Words with Pictures or Gestures

Enrichment by means of pictures is a method that teachers have been using all over the world for a long time. However, the scientific literature on the topic is poor (Cook, 2008). Science has not really focused attention on the reason and extent to which pictures accompanying L2 words support word memorization. In the early 1970s, the use of enriching visual material was explained by the Dual Code Theory (Paivio and Csapo, 1969). According to it, verbal information accompanied by a picture during encoding leads to the formation of a mental image. This image consists of a verbal and a visual code. The codes are processed along different pathways and stored in different domains of the mind. During retrieval, the person can access the word in L2 not only from the word in the native language (L1) but also through the image. If the verbal code decays, the picture can make the word accessible. Recent neuroscientific studies, including one by Mayer et al. (2015), have provided evidence for a possible neural base of the Dual Code Theory. The authors proved that word learning by means of pictures enhances memory compared to only reading and hearing a word. At the level of processing, Mayer et al. (2015) demonstrated that pictures and words are in fact processed and stored in different areas of the brain. Accordingly, concepts are large brain networks that retain the combined information and make it more resistant to decay.

Another influential theory, the Level of Processing Framework (LOP) (Craik and Lockhart, 1972) explains why enrichment by pictures leads to better memory performance. The LOP predicts that memory outcome depends on the degree of depth achieved during information encoding. If a word is encoded in a shallow way, at the phonological level, for example, by only hearing it, the recall of that word will be less efficient than if it were processed deeply by adding sensory information. In this respect, enriching a word with a picture or through other sensory experiences, i.e., touching a referential object or smelling an odor connected to it, etc., makes encoding deeper than only reading the word. Furthermore, semantic processing of the word through questions on its meaning or category and other intrinsic features can make word encoding deep and consequently support memory.

In L2 practice, besides illustrative pictures, pantomime has been used in the classroom for decades (Seaver, 1992). However, here the focus is on self-performed gestures or actions that can accompany novel words during learning and enrich them multisensorily as a learning strategy (Macedonia, 2013). Allen (1995) made English natives learn French phrases with emblematic gestures. Tellier (2008) trained 20 French pre-schoolers with eight English words associated with self-performed gestures and with pictures. Macedonia and Klimesch (2014) had university students learn an artificial corpus audio-visually and with gestures. These studies demonstrated that learners retrieved words learned with gestures significantly better than words encoded audio-visually (for reviews, see Macedonia and von Kriegstein, 2012; Macedonia, 2014).

The use of gestures that support memory for verbal information was originally investigated in action memory research. Starting in the early 1980s, various groups conducted experiments on self-performed action. They showed that self-performance enhances the recall of verbal information compared to either observing an experimenter performing the action or verbal encoding alone (for a review, see Zimmer et al., 2001). The effect of the action/gesture on word retention is referred to as the “enactment effect” (EE) (Engelkamp, 1998). EE is a robust mechanism documented by various memory tests: free recall (Engelkamp and Dehn, 2000; Schatz et al., 2011), cued recall (Kormi-nouri, 1995; Kubik et al., 2014), and word recognition (Engelkamp et al., 1994; Golly-Häring and Engelkamp, 2003).

Various theoretical accounts have explained the enactment effect. Engelkamp and Krumnacker (1980) attributed the retention benefit to a motor trace that the enactment leaves in the word representation. More than two decades after Engelkamp’s seminal publication, studies with brain imaging have demonstrated that acoustic or audio-visual recognition of words encoded with words elicits hemodynamic activity in motor cortices during learning (Nyberg et al., 2001; Masumoto et al., 2006; Eschen et al., 2007). These results empirically validated Engelkamp’s initial intuition. In L2 research, combined behavioral and functional magnet resonance imaging (fMRI) studies have come to similar results. Macedonia et al. (2011) trained participants to learn novel words in L2 with iconic gestures. During brain imaging, word recognition activated a vast language network including motor cortices. Comparable results have demonstrated the role of the motor system in the word’s representation in two recent studies conducted by Mayer et al. (2015, 2017). In these studies participants accessed a word in L2 for a translation task during brain imaging, and motor cortices became active upon word retrieval. Macedonia and Mueller (2016) found activity in further motor structures in the brain, e.g., in the basal ganglia and the cerebellum, during word recognition. Studies in L2 learning have confirmed the involvement of the whole motor system, not only the motor cortices, in word representation. These studies have thus provided further support for the Motor Trace Theory (Engelkamp and Zimmer, 1989). Altogether, brain imaging has brought a new perspective to the representation of word semantics: words in the brain are represented by vast experience-related networks that include the motor acts performed during learning (Hauk et al., 2004; Pulvermüller et al., 2005a). Macedonia and Mueller (2016) further advanced the hypothesis that better memorization occurs because words are stored in both declarative and – because of the gestures – procedural memory.

In the 1980s, EE was also reconducted to motor imagery experienced by the subject (Saltz and Donnenwerth-Nolan, 1981). This is a kinetic image representing the word’s semantics that the learners have in mind when performing the gestures. More than 20 years later, neuroscientific studies conducted on congruency with mismatch paradigms have detected specific neural correlates between words’ semantics and their representational gestures. These studies have demonstrated that we have in mind a motor image representing the word (Kelly et al., 2010). Macedonia et al. (2011) designed their experiment in order to determine whether the memory enhancement comes from the motor trace itself or from the motor image related to the word. They compared the impact of iconic versus meaningless gestures on retrieval of novel words. If the motor component *per se* impacts memory, then the two encoding conditions should yield the same results in memory performance. However, if the benefit comes from the motor image represented by the gestures, then words encoded with iconic gestures should be retrieved better than those encoded with a meaningless gesture. Behavioral data indicated that superior performance was achieved with iconic gestures. Brain imaging revealed that words encoded with iconic gestures activated significantly more the premotor cortices during recognition, whereas words encoded with meaningless gestures involved brain areas related to cognitive control. In other words, participants who learned a novel word with an incongruent gesture displayed a brain activity pattern similar to a Stroop task. In that study (Macedonia et al., 2011), behavioral and imaging data converged to demonstrate that memory performance is not influenced by action execution *per se*; instead, memory is also connected to the internal motor image that the subjects have of a word’s semantics.

Why meaningful gestures support memory can also be explained with theories of embodied cognition. They posit that concepts are stored in the brain together with their perceptual, motor and affective features experienced during encoding (Glenberg, 1997; Barsalou, 2008). A number of studies have shown that in the brain word learning produces experience-related networks (Hauk et al., 2004; Pulvermüller et al., 2005a). Unexpectedly, abstract words also have been found to have embodied representations (Meteyard et al., 2012; Borghi and Zarcone, 2016). When acquiring novel words in L2, learners memorize the “label” to the concept that they embodied previously in their native language (Lupyan and Thompson-Schill, 2012; Yin and Csibra, 2015). However, by only hearing or reading the words, the link between L1 and L2 is weak. It seems that a novel label, the word in L2, needs enrichment in order to be “naturally” stored and easily retrieved. Hence, simply reading the word may deprive our embodied mind of precious input. Using a gesture can re-embody the concept and enact the word’s semantics represented in the motor image.

An important aspect in L2 learning is the speed of word retrieval. In L2, it is of basic importance to have fast access to the vocabulary in order to build sentences and speak the language in real time. Zimmer et al. (2000) observed enhanced efficiency of retrieval in free recall tests if the subjects had enacted the words during study. This “pop-out” effect (POE) was credited to the motor trace created in memory by the actions performed. POE was also found in recognition tasks (Freeman and Ellis, 2003; Daprati et al., 2005) where it was ascribed to low memory load induced by enactment and to better access to information (Spranger et al., 2008). It is speculative, but POE might have to do with the involvement of procedural memory in storage and retrieval of L2 words when they are learned with gestures. Representation in a widely interconnected network could accelerate the speed of retrieval.

#### Abstract Word Learning and Enrichment

Taken together, empirical research has demonstrated that iconic gestures support concrete word learning in L2. Following the embodied approach, abstract concepts can also be grounded in bodily experience, however, in different ways. Whereas concrete nouns and verbs evoke sensory-motor representations (Hauk et al., 2004; Repetto et al., 2013, 2015), abstract words are linked to socio-linguistic information. This has been proposed by the Words as Social Tools Theory by Borghi et al. (2013, 2016). Furthermore, abstract words have an emotional valence, as described by the Affective Embodiment Account (Kousta et al., 2011; Vigliocco et al., 2014).

In L2 abstract word learning, little is known about the effect of enrichment on memorization. Unlike concrete words, abstract words can be controlled for confounding factors inherently. In fact, abstract words do not incorporate strong sensory-motor features in their representations nor are they expected to induce spontaneous visual or motor imagery as concrete words do. To our knowledge, only two studies have explored the effect of enrichment in L2 abstract word learning. Macedonia and Knösche (2011) tested the efficacy of gestures on memory for abstract words in L2. In that study, participants learned 32 abstract sentences consisting of subject, adverb, verb, and object. Only the subject was a concrete noun describing the actor. The other words were abstract. Half of the sentences were encoded audio-visually by reading the sentence and listening to an audio file. The other half were accompanied by self-performed symbolic gestures in addition to the audio-visual encoding. In a 5-day training, participants showed superior memory performance for items encoded with gestures, starting from day 3, however. Additionally, in written sentence production, participants used more items that had been encoded with gestures than items learned audiovisually. In the second study, conducted by Mayer et al. (2017), participants were trained for 4 days on single words that were enriched either by a picture or by a video of an actress performing symbolic gestures. Participants had to perform the gestures themselves and draw the outline of the picture with their right index finger. Memory was assessed by a translation task. Enrichment lead to memory enhancement compared to the baseline (only reading the word and hearing an audio file) but there was no difference in memory performance between the two types of enrichment.

In the present study, we test enrichment on memory for abstract words in L2. We compare enrichment of a written baseline to pictures and gestures. We hypothesize that enrichment makes the word retrieval more accurate and faster compared to a word that is learned without enrichment. However, we hypothesize that the motor component is crucial to enhancing memory compared with the input provided by a picture because of EE. Hence, words encoded with gestures will be better remembered than words encoded with pictures and words encoded with no enrichment. For words encoded with gestures, besides expecting better memory results, we also hypothesize enhanced speed of retrieval in a reaction time task, along with a low error rate. Thus we expect L2 words encoded with gestures, compared to words learned with no enrichment and words learned with pictures, to yield better results in retrieval, evoke POE and be recognized more accurately.

## Materials and Methods

### Participants

Twenty volunteers (12 females; mean age = 30.45, range = 17–47; mean years of education = 17.7, range: 13–21) were recruited within the Psychology Department at the Istituto Auxologico Italiano and by public advertisement. Participants were native Italian speakers with normal or corrected-to-normal vision and no history of neurological or psychiatric diseases. All participants showed normal learning abilities compared to the reference population (mean corrected score for immediate recall = 48.05/75, *SD* = 8; mean corrected score for the delayed recall = 10.08/15, *SD* = 3,41), as assessed by the Rey’s Word List (Carlesimo et al., 1996). None of the participants were aware of the specific purpose of the study. They signed an informed consent in order to take part in the experiment. The experimental protocol had been previously approved by the Catholic University Ethics Committee.

### Stimuli

Thirty Italian abstract words were selected for the experiment. These words indicate concepts related to mental processes, symbolic activities, relations, values, and social constructs (Hoffman, 2016).

Each Italian word was arbitrarily assigned to a word taken from the Vimmi corpus, an artificial vocabulary created for experimental purposes (Macedonia et al., 2011). Each Vimmi word was tri-syllabic and conformed with Italian phonotactics, but was different from the Italian lemmas. The complete set of items, including their translation, is listed in **Table 1** (with English translation in parentheses for readers here).

**Table 1:** List of the stimuli in Italian (English translation) and Vimmi.

Italian (English)	VIMMI
avvertenza (warning)	gubame
pretesto (pretext)	pirumo
designazione (designation)	wefino
esercizio (exercise)	fremeda
alternativa (alternative)	mofibu
mentalità (mentality)	gasima
noia (boredom)	elebo
coraggio (bravery)	wirgonu
fatto (fact)	botufe
metodo (method)	efogi
scopo (goal)	dizela
disdetta (cancelation)	munopa
avanzamento (development)	denalu
sforzo (effort)	utike
correzione (correction)	fapoge
stranezza (oddity)	boruda
cambiamento (change)	zalefa
indifferenza (indifference)	frugazi
autorizzazione (authorization)	frokibe
informazione (information)	sapezo
teoria (theory)	sigule
stile (style)	lifawo
tendenza (trend)	pokute
benessere (well-being)	bekoni
consiglio (advice)	giketa
comando (command)	magosa
tradizione (tradition)	uladi
unione (union)	nabita
terapia (therapy)	giwupo
proprietà (ownership)	mesako

The 30 word pairs were randomly subdivided into three blocks (10 items each) and assigned randomly to one of the three encoding conditions: verbal encoding (VE), picture encoding (PE), and gesture encoding (GE). A series of one-way ANOVAs with word frequency and psycholinguistic parameters as dependent variables confirmed that the words did not differ between conditions (frequency: *F*_(2,27)_ = 1.011; *p* = 0.37; letter length: *F*_(2,27)_ = 3,37; *p* = 0.05; number of syllables: *F*_(2,27)_ = 2.01; *p* = 0.15; orthographic neighborhood: *F*_(2,27)_ = 0.55; *p* = 0.58; phonological neighborhood: *F*_(2,27)_ = 0.97; *p* = 0.39) (De Mauro et al., 1993).

**FIGURE 1:**
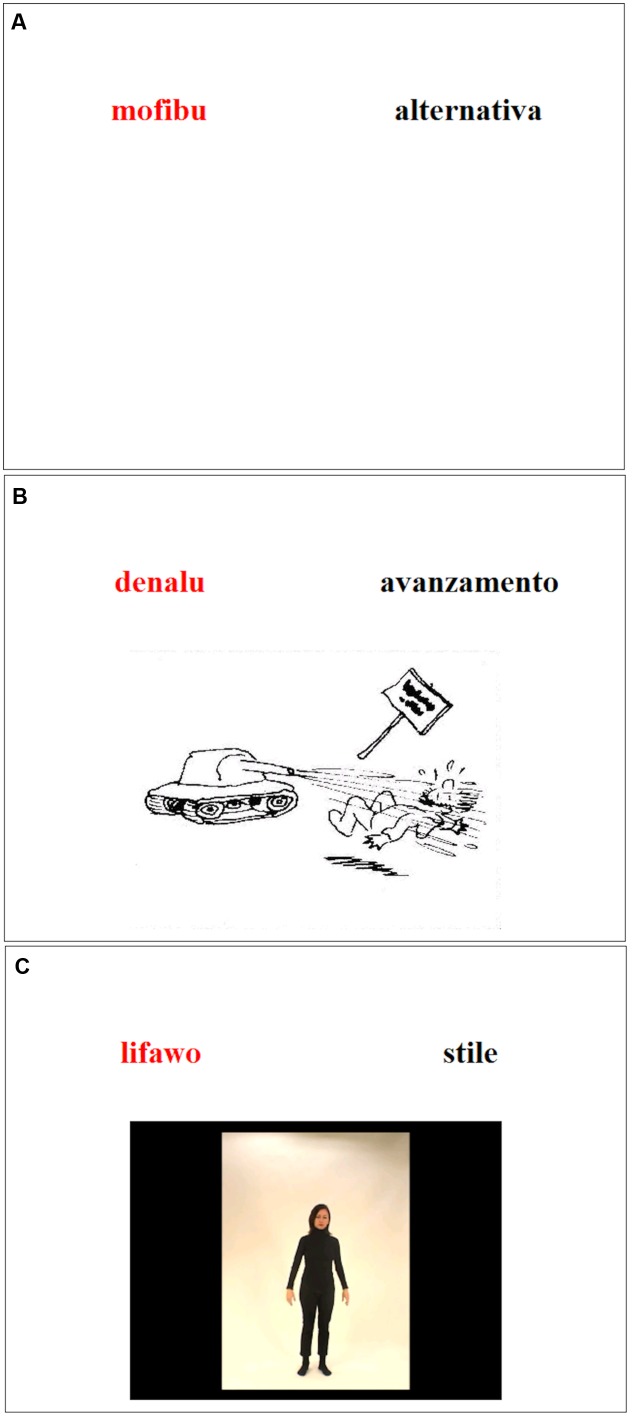
Screenshots of the stimuli in the different encoding conditions: **(A)** VE, **(B)** PE, and **(C)** GE.

In VE, the written words were used. In the PE condition, 10 black-and-white vignettes enriched the words. On the vignettes, a human being performed an action representing the word meaning metaphorically. For example, for the abstract word “effort,” the vignette depicted a cyclist trying to ascend a slope and sweating from the strain. A metaphor refers to one thing by expressing another thing. Here we show the sweating cyclist, but we mean the effort that the cyclist exerts. For the GE condition, 10 video clips were recorded (mean duration: 4.7 s). An actress performed gestures describing the meaning of the abstract words metaphorically. For “tradition,” the actress pantomimed a traditional Tyrolean dance. In most of the clips, the gestures involved mainly the arms and the head. Only one clip also involved the legs. The clips were always structured as follows: the actress started in a standing position, then she performed the gestures once, finally she returned to the standing position. The actual execution of the gestures took about 1,5 to 2 s. We chose metaphoric gestures and discarded meaningless gestures for two reasons. First, previous research has demonstrated that meaningless gestures do not enhance memory (Macedonia et al., 2011; Hupp and Gingras, 2016). Secondly, in order to clarify whether motor execution is crucial in enhancing memory, it was necessary to set up a condition that differed from PE only with regard to the motor component. **Figure 1** represents the screenshots of the stimuli in the different encoding conditions.

### Experimental Procedure

#### Training

The participants were welcomed by a researcher into a quiet room. After reading and signing the informed consent, they began the experimental task. Participants sat in front of a 15^′′^ computer screen at a distance of approximately 50 cm. On the screen, they were presented with the stimuli using E-Prime software [Psychology Software Tools, Inc., E-Prime 2.0 (2012), retrieved from http://www.pstnet.com]. In all conditions, an Italian word appeared in the top left side of the screen in black. It was paired with its Vimmi translation, which appeared in the top right side of the screen in red. Participants were requested to read the Vimmi word aloud. The stimulus slide lasted 6 s. Thereafter, a fixation cross appeared in the center of the screen for 1 s, and the next stimulus was displayed.

**FIGURE 2:**
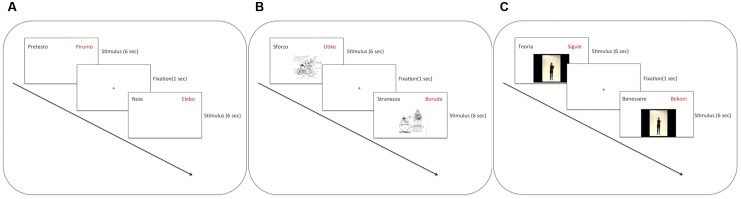
Experimental procedure: **(A)** VE condition, **(B)** PE condition, **(C)** GE condition.

In the VE condition, the stimulus comprises the pair of written words. In the PE condition, a vignette is presented in the middle of the screen below the words. In the GE condition, a video of an actress performing the metaphoric gestures appears in the middle of the screen. In both the PE and the GE conditions, the words and the visual stimuli appeared simultaneously. The video started automatically once the slide appeared on the screen (see **Figure 2** for the timeline of the training in the different experimental conditions). An instruction screen was displayed at the beginning of the tasks: “In this experiment, you will see Vimmi words (red) and their translation into Italian (black) at the top of the screen. Your task is to memorize as many pairs as possible (Vimmi-Italian). You must always repeat the Vimmi word aloud.” Before each training condition, a specific instruction screen was presented. For the VE condition the instruction was: “Read aloud the word in red”; for the PE condition: “Read aloud the word in red and look at the picture”; for the GE condition: “Stand up, read aloud the word in red, watch and perform the gesture.”

The encoding session was split into two parts (which we call “subsessions”) with a short break of about 4 min between sessions. During the break, a collection of static landscapes was presented on the computer screen. In each encoding subsession, five items per condition were trained in blocks. Within the blocks, each item was repeated 10 times. Each subsession consisted of 150 presentation events. The order of the subsessions was balanced across participants. The order of blocks within the training session and the order of items within each block were randomized. The whole training session lasted approximately 35 min.

#### Memory Tests

Immediately after the training session, the participants underwent word recall and word recognition tests. In paper-and-pencil tests, the volunteers were asked to write as many words as they remembered in Italian (free recall Italian). The same procedure was repeated for Vimmi (free recall Vimmi). Thereafter, a sheet with a randomized list of the 30 trained items was presented. Participants were asked to translate them from Italian into Vimmi and, in a second randomized list, from Vimmi into Italian (cued recall Italian to Vimmi and cued recall Vimmi to Italian, respectively). Participants had 5 min to complete each test. The whole test session lasted approximately 20 min.

After the paper-and-pencil tests, the recognition test started. It was conducted using the software E-Prime. Participants were instructed to read the Vimmi word presented in the center of the screen and to select one of the two possible Italian translations placed on the upper left and upper right side of the screen. Participants could choose by pressing the key “Q” or “P” on the keyboard with the left or right index finger, respectively. The position of correct answers was counterbalanced (presented the same number of times on the right and left sides). Correct translations were coupled with incorrect ones, but plausible translations (i.e., the translation of another word learned in the same experimental condition) were also accepted. Reaction times (RTs) and number of errors were also recorded.

### Data Processing and Statistical Analysis

The recall tests were scored by assigning a value ranging from 0 to 1 to each item. For words in Italian, the score 1 was assigned to accurate recall and 0 to the lack of recall. For words in Vimmi, the score 1 was assigned to the accurate recall, 0.5 for an item in which 2 syllables over 3 were correct, 0 for a word with only one syllable matching the correct word or for a missing word. For each test, in each encoding condition the maximum score was 10 and the total maximum score was 30.

For the recognition test, the number of incorrect responses was computed for each participant and for each encoding condition. Incorrect responses were deleted, and the mean RTs for each encoding condition were calculated only on the items that were correctly recognized. **Tables 2**, **3** report the descriptive data by subjects and by items respectively.

**Table 2:** Descriptive data (by subjects).

Memory measure	Encoding	Mean	*SD*	Minimum	Maximum
	condition				
Free recall Vimmi	VE	1.58	1.88	0	5.5
	PE	1.55	1.53	0	5
	GE	1.80	1.96	0	7
Free recall Italian	VE	3.30	2.47	0	9
	PE	4.05	1.90	0	8
	GE	6.55	2.14	2	10
Cued recall Italian to Vimmi	VE	1.73	2.29	0	8
	PE	1.45	1.88	0	7
	GE	2.08	2.45	0	8
Cued recall Vimmi to Italian	VE	3.10	2.95	0	10
	PE	3.10	2.88	0	9
	GE	4.33	2.53	1	10
RTs	VE	1986.34	694.50	869.20	3330.57
	PE	2227.07	700.56	1200.71	3381.00
	GE	2069.21	667.93	986.40	3345.86
Recognition errors	VE	1.70	1.53	0	5
	PE	2.50	1.70	0	5
	GE	1.35	1.39	0	4

**Table 3:** Descriptive data (by items).

Memory test	Encoding	Mean	*SD*	Minimum	Maximum
	condition				
Free recall Vimmi	VE	3.40	1.61	0.00	5.50
	PE	3.40	1.41	1.00	6.00
	GE	4.0	1.52	1.00	6.00
Free recall Italian	VE	6.60	2.11	3.00	10.00
	PE	8.30	2.40	3.00	11.00
	GE	13.10	2.68	9.00	17.00
Cued recall Italian to Vimmi	VE	3.55	3.22	1.00	11.50
	PE	3.20	1.56	1.00	5.50
	GE	4.60	2.79	0.00	8.50
Cued recall Vimmi to Italian	VE	6.60	3.83	1.00	13.00
	PE	6.50	2.95	3.00	11.00
	GE	9.15	3.46	4.00	14.00
RTs	VE	2039.12	354.42	1688.90	2747.68
	PE	2260.96	270.26	1838.26	2715.30
	GE	2045.62	174.77	1811.55	2360.35
Recognition errors	VE	3.40	2.17	0.00	6.00
	PE	5.10	1.52	3.00	8.00
	GE	2.70	1.05	1.00	4.00

The recall scores, the RTs and the number of recognition errors were entered in separated repeated measures ANOVAs by subjects with *encoding condition* as the within-subject variable with three levels (VE, PE, GE). The data were also analyzed by items by means of a series of one-way ANOVAs, with *encoding condition* as the between-subjects variable. *Post hoc* analyses between conditions were computed when needed, with Bonferroni correction for multiple comparisons.

## Results

In the *free recall Italian* test, we found a main effect of encoding condition, both by subjects and by items [F1(2,38) = 22.89; *p* < 0.01; η^2^= 0.54; F2(2,27) = 19.49; *p* < 0.01; η^2^= 0.59]. Specifically, items trained in the GE condition were better remembered than those trained in the PE and VE conditions in both by subjects and by items analyses (*p* < 0.01). However, the performance in the PE and VE conditions did not differ [by participants, *p* = 0.54; by items, *p* = 0.38]. In the *free recall Vimmi* test, the learning condition did not affect memory performance [F1(2,38) = 2.38; *p* = 0.8; F2(2,27) = 0.52; *p* = 0.6].

In the cued recall tests, the data evidenced similar patterns to those found in the free recall tests. In the Vimmi to Italian test, the encoding condition proved to have an impact on the number of recalled items only in the by subjects analysis [F1(2,38) = 5.47; *p* < 0.01; η^2^= 0.22; F2(2,27) = 1.91; *p* = 0.17]. *Post hoc* tests in the by subjects analysis revealed that the GE condition was more effective than PE (*p* = 0.03), whereas PE and VE did not differ (*p* = 1). The difference between GE and PE was almost significant (*p* = 0.05). The Italian to Vimmi test showed similar performance in all of the encoding conditions [F1(2,38) = 1.88; *p* = 0.17; F2(2,27) = 0.77; *p* = 0.47].

We checked the statistical power because of the modest sample size in the present study (*N* = 20). A *post hoc* power analysis conducted with the program G^∗^Power (Faul et al., 2007) revealed that on the basis of the mean, i.e., the within-groups comparison, the effect size observed for the Vimmi to Italian cued recall test (=0.22) and our sample size (*N* = 20) reached statistical power at the recommended level, i.e., 0.80 (Cohen, 1988).

In the recognition test, RTs were affected by the encoding condition in the by subjects but not in the by items analysis [F1(2,38) = 3.8; *p* = 0.03; η^2^= 0.17; F2(2,27) = 2.09; *p* = 0.14]. *Post hoc* tests in the by subjects analysis revealed that participants were slower in recognizing the correct Italian translation if words had been encoded in PE than in the VE condition (*p* < 0.01). The RT of responses did not show significant differences between the PE and GE conditions (*p* = 0.27), nor between GE and VE (*p* = 1). The analyses of the error pattern revealed that there was a main effect of Encoding Condition [F1(2,38) = 3.96; *p* = 0.03; η^2^= 0.17; F2(2,27) = 5.6; *p* < 0.01; η^2^= 0.29]. Participants made less errors in recognizing words trained in the GE than in PE the condition (by subjects, *p* = 0.03; by items, *p* < 0.01), whereas the number of incorrect responses did not differ between the PE and VE conditions (by subjects, *p* = 0.25; by items, *p* = 0.09), nor between GE and VE (*p* = 1).

## Discussion

In the present study, we were interested in understanding whether enrichment through pictures and gestures impacts memory for abstract words. The choice to use this class of words was motivated by the desire to keep sensory-motor information in the representation of the L1 word low in order to observe the potential EE free from influences that might derive from the L1 word semantics. In our within-subjects learning protocol, we asked young adults to memorize 30 foreign abstract words. The words were trained according to three conditions: verbally (VE), by looking at a cartoon representing the word’s semantics (PE), by watching an actress performing a gesture and thereafter by performing the gesture themselves (GE). The analyses conducted both by subjects and by items did not always match across memory outcomes, indicating that the set of stimuli selected might have affected performances. Indeed, in the free recall Italian test did the results show better memory performance for abstract words memorized in association with a gesture, in both the analysis by subjects and by items; in the Cued recall Vimmi to Italian test the beneficial impact of gestures emerged only in the analysis by subjects, indicating that we cannot generalize the results to other sets of words. In the other memory tests, the performance did not differ based on the training condition. In the reaction time test, our hypothesis that gestures would support memory better than pictures was confirmed only partially. In the recognition test, RT values were higher for words encoded with picture enrichment compared to the verbal encoding alone, but again only in the analysis by subjects; however, both the analyses confirmed that accuracy was higher for words learned with gestures than for words encoded with pictures. Also in this case, the results only partially matched our hypothesis. Considering the potential random effect due to the items selection, we will only consider reliable the results that evidenced convergent effects in both the analyses.

Comparing this study with similar studies, i.e., conducted with the same corpus (Vimmi) and stimuli (audio, video files, pictures), population (young adults), the only striking difference resides in training time. It lasted 35 min and can be considered as short in comparison with similar studies. In studies with a robust EE for words (Macedonia and Knösche, 2011; Macedonia et al., 2011) subjects learned much more intensively, i.e., approximately 3 h daily for 5 days. In another combined experiment (behavioral and fMRI) with pictorial and gestural enrichment, gestures proved superior compared to pictures and the audiovisual baseline. This was the case for concrete (Mayer et al., 2015) and for abstract words (Mayer et al., 2017). In the last study, the training lasted about 12 h (subdivided in 4 days, with 3 h daily). Those studies included more items, however. These results seem to indicate that enrichment of a word in L2 with gestures needs longer and repeated training sessions in order to show EE and POE. We speculate that this might involve processes of neuroplasticity affecting procedural learning. As suggested by Macedonia and Mueller (2016), learning novel words with gestures engages procedural memory in addition to declarative memory. In that study, the authors could show that reading and hearing words that had been learned with gestures activate manifold structures of the motor system in the brain. Besides the motor cortices, the basal ganglia and the cerebellum – normally related to motion planning, sequencing and execution – participated in word recognition.

Traditionally, vocabulary learning is situated in the domain of declarative memory (Ullman et al., 1997; Squire and Dede, 2015). However, it is well-demonstrated that word representation in L1 – being experience-related – engages motor structures of the brain (Pulvermüller et al., 2005a,b) at least for those words that in their semantics include some kind of action. Thus we reason that the representation of novel items in L2, because of how they are learned, could also be stored in procedural memory. In other words, considering that the representation of a concept and its label (word) in the brain is an experience-related network, if the network has been created by engaging motor regions, the storage of the concept (word) must have to do with procedural (implicit) memory (Reber, 2013). In Doyon’s two-component model of motor learning (Doyon, 2008), we find immediate and slow motor skill learning. Slow motor learning needs consolidation, i.e., dynamic reorganization in motor networks including – besides the motor cortices – the basal ganglia and the cerebellum (Ungerleider et al., 2002). It could be the case that L2 word acquisition with gestures engages slow motor learning. Similarly, it is conceivable that neurobiological processes driving plasticity need “frequent” stimulus repetition in time in order to produce measurable behavioral effects. As described by Reber (2013), experience-driven changes in the adult sensory cortex occur “slowly.” An extremely changeable sensory cortex that continuously adapts and reorganizes by experience would be instable and lose abilities that have been acquired but that are not required often. According to Reber, practice would accrue implicit learning effects slowly – in our case EE – and make them inflexible. These apparently “negative” characteristics of the procedural system, i.e., slow speed of learning and inflexibility could be the key to better memory performance for L2 words learned with gestures. In fact, studies with massed training over 4 or 5 days show that significant better performance for gesture learning starts from day 2 to day 3 (Macedonia and Knösche, 2011; Macedonia et al., 2011; Mayer et al., 2015) and follow up tests reveal significantly better retrieval over time, i.e., slower information decay, compared to all other experimental conditions (Macedonia and Klimesch, 2014). Thus, procedural learning takes long(er) but information retrieval is superior in speed and resistance to decay.

A study with 30 Vimmi words and three repetitions per word (Macedonia et al., under revision) yielded poor behavioral results comparable to the present study. Participants lying in the fMRI scanner were asked to memorize the Vimmi items by reading, reading and hearing, or by reading, hearing and observing semantically related gestures. Words learned by observing the gestures were significantly better memorized than those learned in the other conditions, however, in the free recall in L1 only. Free recall in Vimmi and cued recalls did not show better performance for gesture observation training. Although participants were lying quietly in the scanner, the brain imaging revealed simulation activity in motor regions triggered by the gesture observation. Still, behavioral performance was low. We attribute this again to the low number of repetitions (3) and to slow motor learning.

Despite the “poor” training in the present study, gestural training enabled participants to perform the “easy” task, i.e., free recall in L1, better than the other training conditions. It is not new that enactment substantially improves performance on conceptually driven explicit-memory tests (Nyberg and Nilsson, 1995), i.e., free recall. We reason that during L2 word learning, the concept is stored first. Thereafter the label to the concept follows as documented in infancy for L1 (Yin and Csibra, 2015). In free recall, when learners are asked to retrieve the words in L2, it is possible that retrieval starts at the conceptual level. Learners know the concepts and label it in L1. However, naming the concepts in L2 requires retrievability of the L2 label. This is only possible after adequate learning and connected consolidation processes. In contrast to infants (Yin and Csibra, 2015) and depending on the age of the learner (Schatz et al., 2010), this process might need more stimulation than participants were provided in the present study. Hence, when learners retrieve the concept’s label, the word in L1 is an easy task compared to retrieving the word in L2. Results showing that retrieval in L1 comes before retrieval in L2 have been previously reported (Macedonia et al., 2011). They have been attributed to the duration of the training in relation to the difficulty of the task. These factors can modulate the effects of gesture-based training as previously addressed in a study by Macedonia et al. (2010) that investigated the impact of gestures on foreign word learning in high and low performers. The former took advantage of gestures only in the more difficult tasks (free recall in L2 and cued recall from L1 into L2), whereas the latter also did so in the easier ones as in the present study. In a similar study yielding poor behavioral results, Krönke et al. (2013) reasoned that gestures can promote learning only when the task can be considered difficult related to the individual’s learning abilities. In the reverse situation, when the task is “easy,” learners can use other cognitive strategies that mask the effect of gestures. Thus, it stands to reason that the impact of gestures on learning is a complex mechanism that emerges from the intersection of various variables, including the difficulty of the task and the duration of the training.

In the recognition task, we had hypothesized short reaction times for GE items as described in the literature (Spranger et al., 2008). Unexpectedly, we found no differences in the RTs among conditions. This result does not match the POE (Zimmer et al., 2000), in which lower reaction times would be expected. Though there might be an explanation for our RT results. Considering the short training and that the recognition test took place after a short break, it might also be the case that learning circuits were not consolidated. If we assume that by the participant performing a gesture while learning a novel word, the information is stored in both declarative and procedural memory, as advanced in Macedonia and Mueller (2016), it stands to reason that the sensorimotor networks need time to consolidate. To this process sleep would also contribute in order to reach full functional efficiency (Brashers-Krug et al., 1996; Stickgold, 2005). Furthermore, we expected that words encoded with gestures would be recognized more accurately than in the other conditions. Actually, participants made less recognition errors with words encoded with gestures than with words encoded with pictures. However, accuracy in recognition did not differ between words encoded with gestures and words encoded with no enrichment.

We hypothesized that besides gestures also pictures (PE) would have a positive impact on memory compared to pure verbal encoding (VE). However, our data did not provide evidence for this hypothesis, and consequently for the Dual Code Theory (Paivio and Csapo, 1969) and the LOP Framework (Craik and Lockhart, 1972). However, we do not question these theories. We speculate that in our study better retrieval effects might have occurred after more intensive training with a higher number of picture presentations.

Taken together, the present findings partially support our prediction: enacted gestures, compared to pictorial enrichment, are critical to enhancing learning. This perspective is coherent with Engelkamp’s approach (Engelkamp, 2001), which assigns the major role to a motor trace derived from the self-performed action. Our position agrees with approaches of embodied cognition (Glenberg, 2007; Barsalou, 2008). We deduce that even if abstract words are represented in the brain with less visual-sensory-motor features than concrete words, superimposing a motor component related to word meaning promotes learning. It is noteworthy that this is not merely a matter of adding information. Even motor enrichment *per se* is not as effective as gesture (see Macedonia et al., 2011; Mayer et al., 2017). This indicates that, in our cognitive system, gesture holds a specific privileged status compared to sensory and simple motor modalities. Furthermore, gestures, in addition to intrinsic motor features, are not independent of manifold perceptual and sensory features even if they represent abstract concepts (Vigliocco et al., 2014). During encoding, gestures enhance verbal memory performance because they also make sensory information connected to the concept available by simulation.

A limitation to this study is that the experimental protocol did not allow us to collect data about consolidated memories and about the decay of memories over time. We cannot provide information on whether gesture-based training is capable of making memory traces more stable than enrichment by pictures. Future research could address this issue by setting up multiple-day training with consolidation phases and successive follow-up tests.

Considering that this research might flow into educational practice, learning of vocabulary items by means of pictures and gestures is recommended particularly in low access-level instruction. Even a few repetitions of enriched L2 words lead to better memory performance and accuracy. However, massed and highly frequent practice by means of enrichment could be the key to exploiting EE and POE and leading to superior memory performance, faster recognition, and boosted retrieval.

## Author Contributions

CR and MM conceived the study. CR and EP set up the experimental protocol, collected and analyzed the data. CR and MM wrote the paper.

## Conflict of Interest Statement

The authors declare that the research was conducted in the absence of any commercial or financial relationships that could be construed as a potential conflict of interest.

## References

[B1] Allen L. Q. (1995). The effects of emblematic gestures on the development and access of mental representations of French expressions. *Modern Lang. J.* 79 521–529. 10.1111/j.1540-4781.1995.tb05454.x

[B2] Barsalou L. W. (2008). Grounded cognition. *Annu. Rev. Psychol.* 59 617–645. 10.1146/annurev.psych.59.103006.093639 17705682

[B3] Borghi A. M. Binkofski F. Castelfranchi C. Cimatti F. Scorolli C. Tummolini L. (2016). The challenge of abstract concepts. *Psychol. Bull.* 143 263–292. 10.1037/bul0000089 28095000

[B4] Borghi A. M. Scorolli C. Caligiore D. Baldassarre G. Tummolini L. (2013). The embodied mind extended: using words as social tools. *Front. Psychol.* 4:214. 10.3389/fpsyg.2013.00214 23641224 PMC3640182

[B5] Borghi A. M. Zarcone E. (2016). Grounding abstractness: abstract concepts and the activation of the mouth. *Front. Psychol.* 7:1498. 10.3389/fpsyg.2016.01498 27777563 PMC5056183

[B6] Brashers-Krug T. Shadmehr R. Bizzi E. (1996). Consolidation in human motor memory. *Nature* 382 252–255. 10.1038/382252a0 8717039

[B7] Carlesimo G. A. Caltagirone C. Gainotti G. Fadda L. Gallassi R. Lorusso S. (1996). The mental deterioration battery: normative data, diagnostic reliability and qualitative analyses of cognitive impairment. *Eur. Neurol.* 36 378–384. 10.1159/000117297 8954307

[B8] Cohen J. (1988). *Statistical Power Analysis for the Behavioral Sciences.* Hillsdale, NJ: Lawrence Erlbaum Associates.

[B9] Cook V. (2008). *Second Language Learning and Language Teaching.* London: Hodder Education.

[B10] Craik F. I. M. Lockhart R. S. (1972). Levels of processing: a framework for memory research. *J. Verb. Learn. Verb. Behav.* 11 671–684. 10.1016/S0022-5371(72)80001-X

[B11] Daprati E. Nico D. Saimpont A. Franck N. Sirigu A. (2005). Memory and action: an experimental study on normal subjects and schizophrenic patients. *Neuropsychologia* 43 281–293. 10.1016/j.neuropsychologia.2004.11.014 15707912

[B12] De Mauro T. Mancini F. Vedovelli M. Voghera M. (1993). *Lessico di Frequenza dell’ Italiano Parlato (LIP).* Milano: Etas libri.

[B13] Doyon J. (2008). Motor sequence learning and movement disorders. *Curr. Opin. Neurol.* 21 478–483. 10.1097/WCO.0b013e328304b6a3 18607210

[B14] Engelkamp J. (1998). *Memory for Actions.* Hove: Psychology Press.

[B15] Engelkamp J. (2001). “Action memory: a system- oriented approach,” in *Memory for Action: A Distinct form of Episodic Memory?* eds Zimmer H. D. Cohen R. L. Guynn M. J. Kormi-Nouri R. Foley M. A. (New York: NY: Oxford University Press), 46–96.

[B16] Engelkamp J. Dehn D. M. (2000). Item and order information in subject-performed tasks and experimenter-performed tasks. *J. Exp. Psychol. Learn. Mem. Cogn.* 26 671–682. 10.1037/0278-7393.26.3.671 10855425

[B17] Engelkamp J. Krumnacker H. (1980). Image- and motor-processes in the retention of verbal materials. *Z. Exp. Angew. Psychol.* 27 511–533.

[B18] Engelkamp J. Zimmer H. D. (1989). Memory for action events: a new filed of research. *Psychol. Res.* 51 153–157. 10.1007/BF00309142 2694204

[B19] Engelkamp J. Zimmer H. D. Mohr G. Sellen O. (1994). Memory of self-performed tasks: self-performing during recognition. *Mem. Cognit.* 22 34–39. 10.3758/BF03202759 8035683

[B20] Eschen A. Freeman J. Dietrich T. Martin M. Ellis J. Martin E. (2007). Motor brain regions are involved in the encoding of delayed intentions: a fMRI study. *Int. J. Psychophysiol.* 64 259–268. 10.1016/j.ijpsycho.2006.09.005 17113672

[B21] Faul F. Erdfelder E. Lang A.-G. Buchner A. (2007). G^∗^Power 3: a flexible statistical power analysis program for the social, behavioral, and biomedical sciences. *Behav. Res. Methods* 39 175–191. 10.3758/BF03193146 17695343

[B22] Freeman J. E. Ellis J. A. (2003). Aging and the accessibility of performed and to-be-performed actions. *Aging Neuropsychol. Cogn.* 10 298–309. 10.1076/anec.10.4.298.28975

[B23] Glenberg A. M. (1997). What memory is for. *Behav. Brain Sci.* 20 1–55. 10.1017/S0140525X97000010 10096994

[B24] Glenberg A. M. (2007). “Language and action: creating sensible combinations of ideas,” in *Oxford Handbook of Psycholinguistics*, ed. Gaskell G. (Oxford: Oxford University Press), 361–370.

[B25] Golly-Häring C. Engelkamp J. (2003). Categorical-relational and order-relational information in memory for subject-performed and experimenter-performed actions. *J. Exp. Psychol. Learn. Mem. Cogn.* 29 965–975. 10.1037/0278-7393.29.5.965 14516228

[B26] Hauk O. Johnsrude I. Pulvermüller F. (2004). Somatotopic representation of action words in human motor and premotor cortex. *Neuron* 41 301–307. 10.1016/S0896-6273(03)00838-9 14741110

[B27] Hoffman P. (2016). The meaning of “life” and other abstract words: insights from neuropsychology. *J. Neuropsychol.* 10 317–343. 10.1111/jnp.12065 25708527 PMC5026063

[B28] Hupp J. M. Gingras M. C. (2016). The role of gesture meaningfulness in word learning. *Gesture* 15 340–356. 10.1075/gest.15.3.04hup

[B29] Kelly S. D. Creigh P. Bartolotti J. (2010). Integrating speech and iconic gestures in a stroop-like task: evidence for automatic processing. *J. Cogn. Neurosci.* 22 683–694. 10.1162/jocn.2009.21254 19413483

[B30] Klimesch W. (1987). A connectivity model for semantic processing. *Psychol. Res.* 49 53–61. 10.1007/BF00309203

[B31] Klimesch W. (1994). *The Structure of Long-Term Memory: A Connectivity Model for Semantic Processing.* Hillsdale, NJ: Lawrence Erlbaum.

[B32] Kormi-nouri R. (1995). The nature of memory for action events: an episodic integration view. *Eur. J. Cogn. Psychol.* 7 337–363. 10.1080/09541449508403103

[B33] Kousta S.-T. Vigliocco G. Vinson D. P. Andrews M. Del Campo E. (2011). The representation of abstract words: why emotion matters. *J. Exp. Psychol. Gen.* 140 14–34. 10.1037/a0021446 21171803

[B34] Krönke K. M. Mueller K. Friederici A. D. Obrig H. (2013). Learning by doing? The effect of gestures on implicit retrieval of newly acquired words. *Cortex* 49 2553–2568. 10.1016/j.cortex.2012.11.016 23357203

[B35] Kubik V. Söderlund H. Nilsson L.-G. Jönsson F. U. (2014). Individual and combined effects of enactment and testing on memory for action phrases. *Exp. Psychol.* 61 347–355. 10.1027/1618-3169/a000254 24503878

[B36] Lupyan G. Thompson-Schill S. L. (2012). The evocative power of words: activation of concepts by verbal and nonverbal means. *J. Exp. Psychol. Gen.* 141 170–186. 10.1037/a0024904 21928923 PMC4124531

[B37] Macedonia M. (2013). Learning a second language naturally: the voice movement icon approach. *J. Educ. Dev. Psychol.* 3 102–116. 10.5539/jedp.v3n2p102

[B38] Macedonia M. (2014). Bringing back the body into the mind: gestures enhance word learning in foreign language. *Front. Psychol.* 5:1467. 10.3389/fpsyg.2014.01467 25538671 PMC4260465

[B39] Macedonia M. Klimesch W. (2014). Long-term effects of gestures on memory for foreign language words trained in the classroom. *Mind Brain Educ.* 8 74–88. 10.1111/mbe.12047

[B40] Macedonia M. Knösche T. R. (2011). Body in mind: how gestures empower foreign language learning. *Mind Brain Educ.* 5 196–211. 10.1111/j.1751-228X.2011.01129.x

[B41] Macedonia M. Mueller K. (2016). Exploring the neural representation of novel words learned through enactment in a word recognition task. *Front. Psychol.* 7:953. 10.3389/fpsyg.2016.00953 27445918 PMC4923151

[B42] Macedonia M. Müller K. Friederici A. D. (2010). Neural correlates of high performance in foreign language vocabulary learning. *Mind Brain Educ.* 4 125–134. 10.1111/j.1751-228X.2010.01091.x

[B43] Macedonia M. Muller K. Friederici A. D. (2011). The impact of iconic gestures on foreign language word learning and its neural substrate. *Hum. Brain Mapp.* 32 982–998. 10.1002/hbm.21084 20645312 PMC6870319

[B44] Macedonia M. von Kriegstein K. (2012). Gestures enhance foreign language learning. *Biolinguistics* 6 393–416. 10.3389/fpsyg.2014.01467 25538671 PMC4260465

[B45] Masumoto K. Yamaguchi M. Sutani K. Tsuneto S. Fujita A. Tonoike M. (2006). Reactivation of physical motor information in the memory of action events. *Brain Res.* 1101 102–109. 10.1016/j.brainres.2006.05.033 16782071

[B46] Mayer K. M. Macedonia M. von Kriegstein K. (2017). Recently learned foreign abstract and concrete nouns are represented in distinct cortical networks similar to the native language. *Hum. Brain Mapp.* 38 4398–4412. 10.1002/hbm.23668 28580681 PMC6867000

[B47] Mayer K. M. Yildiz I. B. Macedonia M. Von Kriegstein K. (2015). Visual and motor cortices differentially support the translation of foreign language words. *Curr. Biol.* 25 530–535. 10.1016/j.cub.2014.11.068 25660537

[B48] Meteyard L. Cuadrado S. R. Bahrami B. Vigliocco G. (2012). Coming of age: a review of embodiment and the neuroscience of semantics. *Cortex* 48 788–804. 10.1016/j.cortex.2010.11.002 21163473

[B49] Nyberg L. Nilsson L. G. (1995). The role of enactment in implicit and explicit memory. *Psychol. Res.* 57 215–219. 10.1007/BF00431282 7753951

[B50] Nyberg L. Petersson K. M. Nilsson L.-G. Sandblom J. Åberg C. Ingvar M. (2001). Reactivation of motor brain areas during explicit memory for actions. *Neuroimage* 14 521–528. 10.1006/nimg.2001.0801 11467924

[B51] Oxford R. Crookall D. (1990). Vocabulary learning: a critical analysis of techniques. *TESL Canada J.* 7 9–30. 10.18806/tesl.v7i2.566

[B52] Paivio A. Csapo K. (1969). Concrete image and verbal memory codes. *J. Exp. Psychol.* 80 279–285. 10.1037/h0027273

[B53] Pulvermüller F. Hauk O. Nikulin V. V. Ilmoniemi R. J. (2005a). Functional links between motor and language systems. *Eur. J. Neurosci.* 21 793–797. 10.1111/j.1460-9568.2005.03900.x 15733097

[B54] Pulvermüller F. Shtyrov Y. Ilmoniemi R. (2005b). Brain signatures of meaning access in action word recognition. *J. Cogn. Neurosci.* 17 884–892. 10.1162/0898929054021111 15969907

[B55] Reber P. J. (2013). The neural basis of implicit learning and memory: a review of neuropsychological and neuroimaging research. *Neuropsychologia* 51 2026–2042. 10.1016/j.neuropsychologia.2013.06.019 23806840

[B56] Repetto C. Colombo B. Cipresso P. Riva G. (2013). The effects of rTMS over the primary motor cortex: the link between action and language. *Neuropsychologia* 51 8–13. 10.1016/j.neuropsychologia.2012.11.001 23142706

[B57] Repetto C. Colombo B. Riva G. (2015). *Is Motor Simulation Involved During Foreign Language Learning? A Virtual Reality Experiment.* Newcastle upon Tyne: SAGE 10.1177/2158244015609964

[B58] Riesenberg L. A. Leitzsch J. Little B. W. (2009). Systematic review of handoff mnemonics literature. *Am. J. Med. Qual.* 24 196–204. 10.1177/1062860609332512 19269930

[B59] Saltz E. Donnenwerth-Nolan S. (1981). Does motoric imagery facilitate memory for sentences? A selective interference test. *J. Verbal Learn. Verbal Behav.* 20 322–332. 10.1016/S0022-5371(81)90472-2

[B60] Schatz T. R. Spranger T. Knopf M. (2010). Is there a memory profit after repeated learning of subject-performed actions? Comparing direct and long-term memory performance level as a function of age. *Scand. J. Psychol.* 51 465–472. 10.1111/j.1467-9450.2010.00828.x 20546198

[B61] Schatz T. R. Spranger T. Kubik V. Knopf M. (2011). Exploring the enactment effect from an information processing view: what can we learn from serial position analyses? *Scand. J. Psychol.* 52 509–515. 10.1111/j.1467-9450.2011.00893.x 21605121

[B62] Seaver P. W. (1992). Pantomime as an L2 classroom strategy. *Foreign Lang. Ann.* 25 21–31. 10.1111/j.1944-9720.1992.tb00509.x

[B63] Shams L. Seitz A. R. (2008). Benefits of multisensory learning. *Trends Cogn. Sci.* 12 411–417. 10.1016/j.tics.2008.07.006 18805039

[B64] Spranger T. Schatz T. R. Knopf M. (2008). Does action make you faster? A retrieval-based approach to investigating the origins of the enactment effect. *Scand. J. Psychol.* 49 487–495. 10.1111/j.1467-9450.2008.00675.x 18705671

[B65] Squire L. R. Dede A. (2015). Conscious and unconscious memory systems. *Cold Spring Harb. Perspect. Biol* 7:a021667. 10.1101/cshperspect.a021667 25731765 PMC4355270

[B66] Stickgold R. (2005). Sleep-dependent memory consolidation. *Nature* 437 1272–1278. 10.1038/nature04286 16251952

[B67] Tellier M. (2008). The effect of gestures on second language memorisation by young children. *Gesture* 8 219–235. 10.1075/gest.8.2.06tel

[B68] Ullman M. T. Corkin S. Coppola M. Hickok G. Growdon J. H. Koroshetz W. J. (1997). A neural dissociation within language: evidence that the mental dictionary is part of declarative memory, and that grammatical rules are processed by the procedural system. *J. Cogn. Neurosci.* 9 266–276. 10.1162/jocn.1997.9.2.266 23962016

[B69] Ungerleider L. G. Doyon J. Karni A. (2002). Imaging brain plasticity during motor skill learning. *Neurobiol. Learn. Mem.* 78 553–564. 10.1006/nlme.2002.4091 12559834

[B70] Vigliocco G. Kousta S. T. Della Rosa P. A. Vinson D. P. Tettamanti M. Devlin J. T. (2014). The neural representation of abstract words: the role of emotion. *Cereb. Cortex* 24 1767–1777. 10.1093/cercor/bht025 23408565

[B71] Yin J. Csibra G. (2015). Concept-based word learning in human infants. *Psychol. Sci.* 26 1316–1324. 10.1177/0956797615588753 26195636 PMC4641314

[B72] Zimmer H. D. Cohen R. L. Guynn M. J. Engelkamp J. Kormi-Nouri R. Foley M. A. (2001). *Memory for Action: A Distinct form of Episodic Memory?* New York, NY: Oxford University Press.

[B73] Zimmer H. D. Helstrup T. Engelkamp J. (2000). Pop-out into memory: a retrieval mechanism that is enhanced with the recall of subject-performed tasks. *J. Exp. Psychol. Learn. Mem. Cogn.* 26 658–670. 10.1037/0278-7393.26.3.658 10855424

